# Publisher Correction: Divergent SARS-CoV-2 variant emerges in white-tailed deer with deer-to-human transmission

**DOI:** 10.1038/s41564-022-01298-3

**Published:** 2022-12-12

**Authors:** Bradley Pickering, Oliver Lung, Finlay Maguire, Peter Kruczkiewicz, Jonathon D. Kotwa, Tore Buchanan, Marianne Gagnier, Jennifer L. Guthrie, Claire M. Jardine, Alex Marchand-Austin, Ariane Massé, Heather McClinchey, Kuganya Nirmalarajah, Patryk Aftanas, Juliette Blais-Savoie, Hsien-Yao Chee, Emily Chien, Winfield Yim, Andra Banete, Bryan D. Griffin, Lily Yip, Melissa Goolia, Matthew Suderman, Mathieu Pinette, Greg Smith, Daniel Sullivan, Josip Rudar, Oksana Vernygora, Elizabeth Adey, Michelle Nebroski, Guillaume Goyette, Andrés Finzi, Geneviève Laroche, Ardeshir Ariana, Brett Vahkal, Marceline Côté, Allison J. McGeer, Larissa Nituch, Samira Mubareka, Jeff Bowman

**Affiliations:** 1 National Centre for Foreign Animal Disease, Canadian Food Inspection Agency, Winnipeg, Manitoba Canada; 2 Department of Veterinary Microbiology and Preventative Medicine, College of Veterinary Medicine, Iowa State University, Ames, IA USA; 3 Department of Medical Microbiology and Infectious Diseases, University of Manitoba, Winnipeg, Manitoba Canada; 4 Department of Biological Sciences, University of Manitoba, Winnipeg, Manitoba Canada; 5 Faculty of Computer Science, Dalhousie University, Halifax, Nova Scotia Canada; 6 Department of Community Health & Epidemiology, Dalhousie University, Halifax, Nova Scotia Canada; 7 Shared Hospital Laboratory, Toronto, Ontario Canada; 8 Sunnybrook Research Institute, Toronto, Ontario Canada; 9 Wildlife Research and Monitoring Section, Ontario Ministry of Natural Resources and Forestry, Peterborough, Ontario Canada; 10 Ministère des Forêts, de la Faune et des Parcs, Quebec City, Quebec Canada; 11 Public Health Ontario, Toronto, Ontario Canada; 12 Department of Microbiology & Immunology, Western University, London, Toronto, Ontario Canada; 13 Canadian Wildlife Health Cooperative, Ontario-Nunavut, Department of Pathobiology, University of Guelph, Guelph, Ontario Canada; 14 Public Health, Health Protection and Surveillance Policy and Programs Branch, Ontario Ministry of Health, Toronto, Ontario Canada; 15 Centre de Recherche du CHUM, Montréal, Quebec Canada; 16 Département de Microbiologie, Infectiologie et Immunologie, Université de Montréal, Montréal, Quebec Canada; 17 Department of Biochemistry, Microbiology and Immunology, University of Ottawa, Ottawa, Ontario Canada; 18 Ottawa Institute of Systems Biology, University of Ottawa, Ottawa, Ontario Canada; 19 Centre for Infection, Immunity, and Inflammation, University of Ottawa, Ottawa, Ontario Canada; 20 Sinai Health System, Toronto, Ontario Canada; 21 Department of Laboratory Medicine and Pathobiology, University of Toronto, Toronto, Ontario Canada; 22 Environmental and Life Sciences Graduate Program, Trent University, Peterborough, Ontario Canada

**Keywords:** SARS-CoV-2, Evolution

Correction to: *Nature Microbiology* 10.1038/s41564-022-01268-9, published online 10 November 2022.

In the version of this article initially published, Bradley Pickering, Oliver Lung and Finlay Maguire were not denoted as equally contributing to the work, and Samira Mubareka and Jeff Bowman were not denoted as jointly supervising the work. The changes have been made in the HTML and PDF versions of the article.

